# Account of Diseases in an Eastern District of London, from the 20th of November to the 20th of December, 1801

**Published:** 1802-01

**Authors:** 


*Account of Diseases in an Eastern Diſtrict of London, from the 20th of November to the 20th of December,* 1801.

| | |
|---|---|
| ACUTE DISEASES. | |
| Typhus | 7 |
| Peripneumonia | 3 |
| Pleuritis | 2 |
| Dyſenteria | 6 |
| Cholera Morbus | 2 |
| Rheumatiſmus Acutus | 3 |
| CHRONIC DISEASES. | |
| Peripneumonia Notha | 4 |
| Tuſſis | 20 |
| Dyſpnœa | 15 |
| Tuſſis cum Dyſpnœa | 10 |
| Raucedo | 3 |
| Pleurodyne | 4 |
| Phthiſis Pulmonalis | 2 |
| Aſthma | 1 |
| Hydrothorax | 1 |
| Anaſarca | 2 |
| Vertigo | 5 |
| Cephalalgia | 7 |
| Ophthalmia | 2 |
| Epiſtaxis | 2 |
| Otorrhœa | 1 |
| Hæmorrhois | 4 |
| Chloroſis | 3 |
| Amenorrhœa | 7 |
| Dyſmenorrhœa | 2 |
| Vomitus | 3 |
| Rheumatiſmus Chronicus | 20 |
| PUERPERAL DISEASES. | |
| Menorrhagia Lochialis | 3 |
| Enureſis | 1 |
| Rhagas Papilæ | 3 |
| Dolores Poſt Partum | 2 |
| INFANTILE DISEASES. | |
| Aphthæ | 4 |
| Vermes | 3 |
| Diarrhœa | 2 |
| Pertuſſis | 4 |

In the hiſtory of the diſeaſes referred to in the foregoing liſt, nothing has occurred that deſerves particular notice. Thoſe complaints which moſt commonly prevail in the autumnal months are now ſubſiding, and are ſucceeded by different affections of the pulmonary ſyſtem, which generally conſtitute a large proportion of the liſt at this ſeaſon of the year. The ſeverity of froſt has occurred more early than uſual, and has increaſed the number, and aggravated the ſymptoms of thoſe complaints which have their ſeat in the cheſt; they have yielded however in general, to the common mode of treatment, aſſiſted by an attention to regimen and diet.

